# Corticospinal neuron subpopulations with opposing dynamics converge to shape spinal interneuron computations

**DOI:** 10.64898/2026.10.21.760744

**Published:** 2026-10-24

**Authors:** Michelle Sánchez Rivera, Yuxiao Li, Anan Li, Constantinos Eleftheriou, Roman Baravalle, Anand S. Kulkarni, Phillip D. Chapman, Andrés F. Cadena, Francisco J. Alvarez, Jay B. Bikoff, Salvador Dura-Bernal, Xiangning Li, Ian Duguid

## Abstract

Corticospinal neuron (CSN) activation plays a crucial role in orchestrating goal-directed actions, but the functional role of reduced CSN output, a feature conserved across species, remains unknown. By combining population imaging, projection mapping, and modelling in mice performing a Go/NoGo lever-push task, we reveal that distinct corticospinal neuron subpopulations with opposing dynamics (i.e. increased or decreased activity) are selectively associated with skilled movement execution. Both activity profiles are observed throughout CSN somatodendritic compartments and are spatially organized, with increased activity concentrated in upper layer 5B and decreased activity localized to deeper subregions. Notably, single-neuron projection mapping revealed that opposing CSN signals converge onto shared spinal interneurons, while modelling showed that reduced CSN output drives subtractive modulation of spinal interneuron input-output transformations, avoiding output saturation and enabling flexible scaling of output firing rates. These findings redefine corticospinal function by demonstrating that reduced output in a subpopulation of CSNs is critical for shaping spinal cord computations during skilled movement execution.

